# Elucidation of potential sites for antibody engineering by fluctuation editing

**DOI:** 10.1038/s41598-017-10246-9

**Published:** 2017-08-30

**Authors:** Saeko Yanaka, Yoshitaka Moriwaki, Kouhei Tsumoto, Kenji Sugase

**Affiliations:** 1 Bioorganic Research Institute, Suntory Foundation for Life Sciences, Kyoto, Japan; 2 Department of Biotechnology and Agricultural Bioinformatics Research Unit, Graduate School of Agricultural and Life Sciences, The University of Tokyo, Tokyo, Japan; 3 Department of Bioengineering, Graduate School of Engineering, The University of Tokyo, Tokyo, Japan; 4 Laboratory of Medical Proteomics, Institute of Medical Science, The University of Tokyo, Tokyo, Japan; 5 Institute for Molecular Science and Okazaki Institute for Integrative Biosciences, National Institutes of Natural Sciences, Tokyo, Japan; 6 Present Address: Department of Molecular Engineering, Graduate School of Engineering, Kyoto University, Kyoto, Japan

## Abstract

Target-specific monoclonal antibodies can be routinely acquired, but the sequences of naturally acquired antibodies are not always affinity-matured and methods that increase antigen affinity are desirable. Most biophysical studies have focused on the complementary determining region (CDR), which directly contacts the antigen; however, it remains difficult to increase the affinity as much as desired. While strategies to alter the CDR to increase antibody affinity are abundant, those that target non-CDR regions are scarce. Here we describe a new method, designated fluctuation editing, which identifies potential mutation sites and engineers a high-affinity antibody based on conformational fluctuations observed by NMR relaxation dispersion. Our data show that relaxation dispersion detects important fluctuating residues that are not located in the CDR and that increase antigen–antibody affinity by point mutation. The affinity-increased mutants are shown to fluctuate less in their free form and to form a more packed structure in their antigen-bound form.

## Introduction

An increasing number of antibodies are now in therapeutic use^1^. Antibodies can be easily acquired by immunization of model animals and humanization but, because their amino acid sequences are optimized by random shuffling, the resulting affinities are not fully matured against the target molecules^2^. In terms of therapeutic use, there is a large demand for higher-affinity antibodies that enable the dose and cost to be minimized. It is thus important both to design antibodies with higher affinity and to elucidate the detail of antibody–antigen interactions in order to establish a basis for optimizing antibodies.

To improve our understanding of antibody–antigen interactions, many biophysical studies, including crystallographic, kinetic, and thermodynamic analyses, have characterized the events that occur at the antibody–antigen interface^3–7^. Based on the results of such studies, a large number of antibody mutants have been designed and tested^8^. Most of the studies aiming to increase the affinity of an antibody have focused on residues at the antibody–antigen interface, known as the complementary determining region (CDR)^3–7^. This strategy is reasonable because antibodies in nature change their specificity and affinity by randomly mutating the amino acid sequences of the CDR. In particular, the technique of molecular evolution, or phage display, has proved to be successful in affinity maturation of the CDR^6^. Computer-assisted structure-based affinity optimization has also been successful^9^. Therefore, several strategies are available to increase the affinity by modifying the CDR. However, it is difficult to increase the affinity solely by changing the CDR residues^10^ because a CDR sequence is relatively well optimized by nature itself, and other strategies to design antibodies with higher affinity are desirable^2^. One promising strategy might be to regulate the antigen-binding process of an antibody by introducing a mutation in a non-CDR region. The variable domains of the heavy chain (V_H_) and light chain (V_L_) represent potential target regions for mutation because they are sequentially and spatially connected to the CDR. At present, however, there is no established rational method that can specifically identify residues appropriate for mutation in non-CDR regions to increase the affinity to antigen.

Here, we focused on the process of dynamic binding between an antibody and its antigen in order to rationally design an antibody with higher affinity. We considered that many residues in non-CDR regions that do not directly interact with the antigen potentially contribute to the process of antibody–antigen complex formation via conformational fluctuation. Elucidating the mechanism by which these residues contribute to the binding process would provide the basis of a strategy to regulate the antibody–antigen interaction; however, the conformational fluctuations that occur in the antibody–antigen binding process are poorly understood.

A problem in attempting to regulate the conformational fluctuation of an antibody is how to choose the residues to be mutated. Without specific criteria, there are nearly 200 candidate residues in non-CDR regions that might be mutated. Here, we describe a method to identify conformational fluctuations that are relevant to the antigen-binding process. Fluctuations of an antibody can be measured by relaxation dispersion—an NMR method that quantitates conformational exchange rates on the millisecond timescale^11^, which applies to many biologically relevant fluctuations such as ligand-binding and folding^12^. We devised a scheme to select residues for mutation on the basis of relaxation dispersion data. We used the Fv fragment (the hypervariable region comprising the V_H_ and V_L_ domains) of an anti-lysozyme antibody, HyHEL-10^13, 14^, to establish the method. The Fv fragment of HyHEL-10 is easy to overexpress in *Escherichia coli*, and its structure, thermodynamics, and kinetics are well characterized^3, 4, 13, 15^.

We demonstrate that the mutation of fluctuating residues that are not located at the antigen–antibody interaction interface can lead to increased affinity of the antibody. In accordance with our method, we selected only eight candidate residues for mutation and obtained two antibodies with increased antigen affinity. To validate whether the mutations altered the fluctuations of the antibody as anticipated, we measured relaxation dispersions of the two mutants and also applied molecular dynamics simulation to quantify the fluctuations on the nanosecond timescale. Our findings indicate that the mutants have a tight packing structure in the bound form, accounting for their increased antigen affinity.

## Results

### Strategy to identify potential mutation sites based on conformational fluctuation

**Figure 1:**
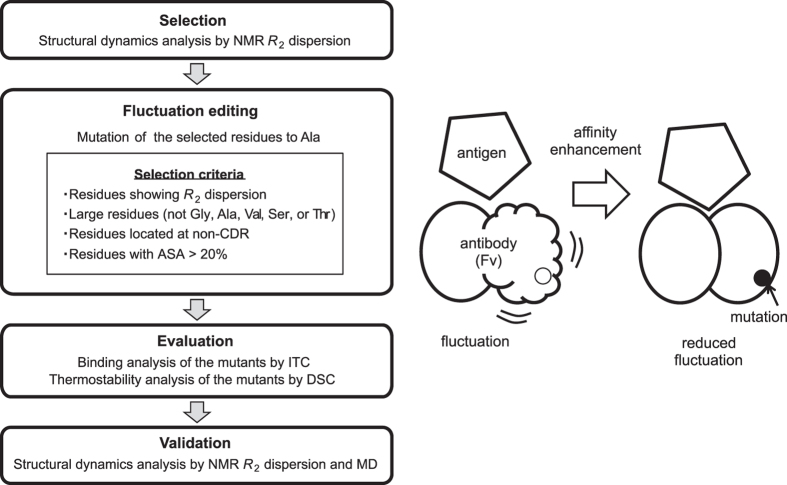
Overview of the fluctuation editing. The experimental strategy to increase the affinity of an antibody and the criteria to select candidate residues for mutation are depicted schematically.

To increase the affinity of an antibody by editing its conformational fluctuations, we devised the following scheme to introduce a mutation at a specific fluctuating residue as shown in Fig. 1 Selection: Conformational fluctuations of an antibody in the free and bound forms are measured by relaxation dispersion. Fluctuation editing: Residues for which a mutation may possibly change the fluctuation are chosen according to the following four criteria: displays relaxation dispersion; located at a non-CDR region; large size (i.e., residues other than Ala, Gly, Ser, Thr, or Val); accessible surface area (ASA) larger than 20%. These criteria are based on the assumptions that mutation of a large fluctuating residue to a smaller residue (Ala) will effectively change the local fluctuation around it, and that residues with a small ASA, or a large buried surface area, will be important for folding of the antibody and should be avoided. Evaluation: Affinity, stability, and fluctuations of the mutants are examined to judge whether the mutants are better than the wild type as anticipated. Validation: To verify the changes in conformation fluctuation of the mutants, relaxation dispersion and MD simulation are conducted.

### Selection: Analysis of fluctuations of HyHEL-10 in the free and lysozyme-bound forms

**Figure 2:**
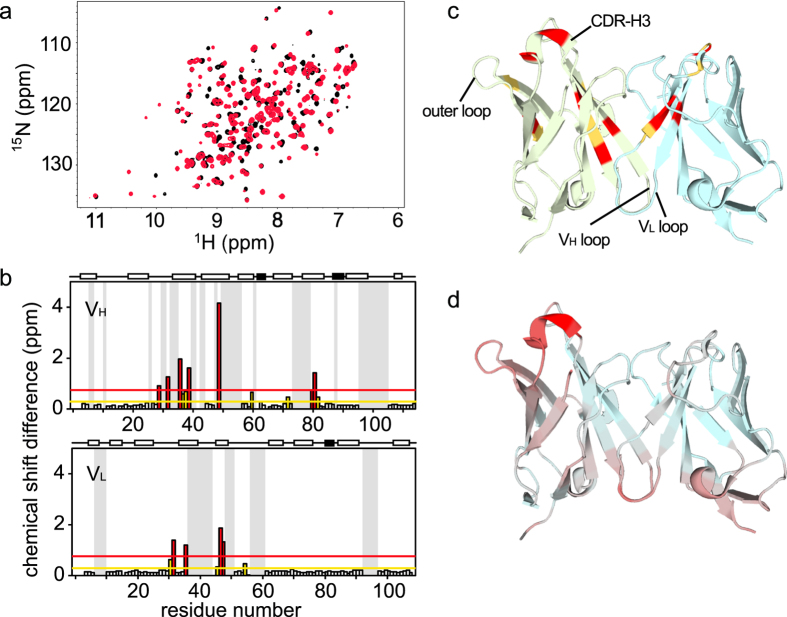
The HyHEL-10 Fv fragment changes its structure upon binding to lysozyme. (**a**) HSQC spectra of free and bound HyHEL-10 are shown in black and red, respectively. (**b**) Chemical shift differences between the free and bound forms are plotted for the V_H_ (top) and V_L_ (bottom) domains. Above each panel, the α-helix and β-strand regions are shown as open and filled boxes, respectively. Yellow and red lines represent the average and average plus one standard deviation of the chemical shift differences, respectively. (**c**) The crystal structure of bound HyHEL-10 (PDBID: 1C08) with residues highlighted in the same colors as in (**b**). The V_H_ and V_L_ domains are shown in light green and light blue, respectively. (**d**) The RMSD between the crystal structure of the free (PDBID: 5AYU) and bound (PDBID: 1C08) forms of HyHEL-10, which are superimposed via the V_L_ domain, is shown in a continuous color scheme from light blue to red, corresponding to RMSD values of 0.08 and 3.27, respectively. Crystal packing that possibly affects the RMSD values was observed in both the 5AYU and 1C08 structures particularly for residues in the crystal packing contact surface at the loops in both V_L_ and V_H_, corresponding to the region colored in red.

First, we measured the^1^H-^15^N heteronuclear single quantum coherence (HSQC) spectra of the free and lysozyme-bound forms of HyHEL-10 (Fig. 2a). Chemical shift differences between the two spectra were mainly observed in the V_H_ domain, especially at the contact surface of the V_H_ and V_L_ domains, and in the outer loop of the V_H_ domain (Fig. 2b,c). This is consistent with the structural differences between the free and lysozyme-bound HyHEL-10 structures (Fig. 2d). The relative orientation of the V_H_ and V_L_ domains differs between the free and bound crystal structures. Each individual domain in the free and bound structures is superimposable with a root-mean-square deviation (RMSD) of 0.32 Å for the V_H_ domain and 0.25 Å for the V_L_ domain. By contrast, the other domain not used for superimposition differs substantially. Both the crystal structures and NMR spectra indicate that the V_H_ domain undergoes a more obvious structural change upon binding to lysozyme.

**Figure 3:**
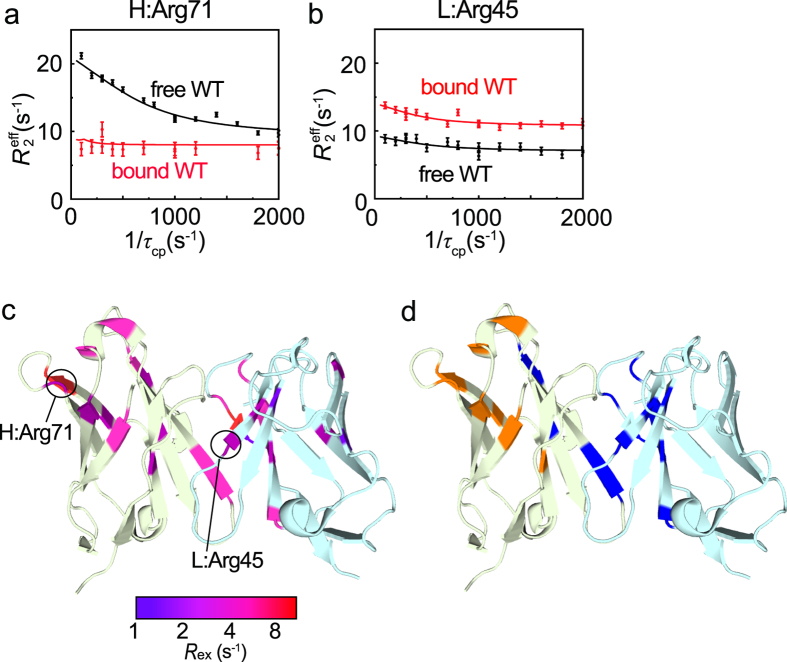
HyHEL-10 fluctuates in its free form. (**a**,**b**) Relaxation dispersion profiles of H:Arg71 (**a**) and L:Arg45 (**b**) at 17.6 T. (**c**) Amplitude of the *R*_ex_ rate mapped on the crystal structure of free HyHEL-10, as a continuous color scheme from purple to red. (**d**) The two clusters fluctuating at the *k*_ex_ rate of 840 ± 15 (blue) and 2033 ± 50 s^−1^ (orange).

Next, we conducted *R*_2_ relaxation dispersion experiments for free HyHEL-10, and observed relaxation dispersions in a wide area of the V_H_ and V_L_ domains (Fig. 3a–c). The fluctuating residues could be divided into two groups (Fig. 3d): one fluctuating at a *k*_ex_ rate of 840 ± 15 s^−1^, and one fluctuating at 2033 ± 50 s^−1^. This result indicates that these regions transiently interconvert with different conformations in solution. Note that the V_H_ and V_L_ domains maintained a tight complex (Fv fragment) during the *R*_2_ dispersion measurements because neither of the domains can exist alone. However, it is possible that local inter-domain interactions were transiently broken and reformed, and this type of process may have contributed to *R*_2_ dispersions. Because the V_H_ and V_L_ domains exist only as a complex, such processes can be treated as internal motions of a single molecule. In Fig. 3c, the amplitude of the relaxation dispersion curve, *R*_ex_, which indicates an excess contribution to the transverse relaxation rate *R*_2_ caused by conformational exchange, is mapped on the crystal structure of free HyHEL-10. Interestingly, most of the relaxation dispersions observed for free HyHEL-10 disappeared when HyHEL-10 formed a complex with lysozyme as typically shown for H:R71 in Fig. 3a. This result suggests that binding to lysozyme stabilizes HyHEL-10. Because all of the *R*_ex_ values were very small, we were unable to determine the conformational exchange parameters precisely. Nevertheless, the smaller *R*_ex_ values in the bound form imply that mutation of the fluctuating residues of free HyHEL-10 might change the fluctuation favorably for lysozyme binding.

### Fluctuation editing: Introducing mutation

Among 32 fluctuating residues identified in free HyHEL-10, small residues and residues buried in the hydrophobic interior with ASA values smaller than 20% were excluded from mutation according to the scheme shown in Fig. 1. The remaining eight residues possessed large side chains and were exposed to the solvent (Supplementary Fig. 1). Each of the eight residues was mutated to alanine, with the expectation that the mutation would change fluctuations around this residue, leading to increase the affinity (Supplementary Table 1).

### Evaluation: Affinity and stability of the mutants

We attempted to express recombinant proteins of all eight antibody mutants, and obtained four mutants in sufficient quantity for subsequent analyses: H:Q3A, H:R44A, H:R71A, and L:R45A (Supplementary Fig. 1), where H and L correspond to the V_H_ and V_L_ domains, respectively. Other mutants could not be obtained due to improper folding during the refolding step. First, the affinity of the mutants for lysozyme was measured by isothermal titration calorimetry (ITC). The H:R44A mutant aggregated during the ITC experiment, and thus was not further analyzed. The association constant *K*_A_ of the H:Q3A mutant ((2.2 ± 0.25) × 10^8^ M) was little changed from that of the wild type^14^ (WT; 1.8 × 10^8^ M). However, this mutant was not stable enough for further fluctuation analysis. In addition to the eight mutants, we also conducted a control ITC measurement for L:H34A, which has a mutation at one of the excluded residues (L:H34). As a result, its affinity was little changed from that of WT ((2.8 ± 0.51) × 10^8^ M), which supports the concept of fluctuation editing.

By contrast, the H:R71A and L:R45A mutants had *K*_A_ values that were more than 10 times higher than that of WT ((2.0 ± 0.88) × 10^9^ M and (2.0 ± 0.46) × 10^9^ M, respectively) (Supplementary Fig. 2). For H:R71A, a higher enthalpy change (Δ*H = *−99.50 ± 0.25 kJ/mol), compensating the loss of entropy (*T*Δ*S = *−47.33 ± 1.07 kJ/mol), was the driving force of the increased affinity: Δ*H = *−78.2 kJ/mol and *T*Δ*S = *−32.2 kJ/mol in the case of WT. The higher enthalpy change suggests the formation of new interactions in the mutant. For L:R45A, a decrease in the loss of entropy (*T*Δ*S = *−16.71 ± 0.56 kJ/mol), compensating the loss of enthalpy (Δ*H = *−68.88 ± 0.21 kJ/mol), contributed to the increased affinity. Further ITC analyses measured at different temperatures showed that the heat capacity change Δ*C*_*p*_ for binding was −2.85 ± 0.08 and −1.04 ± 0.03 kJ/mol/K for H:R71A and L:R45A, respectively (Table 1). For WT, the Δ*C*_*p*_ for lysozyme binding was −1.4 kJ/mol/K^13^. Because a negative Δ*C*_*p*_ value represents dehydration from hydrophobic residues upon binding^16^, these results show that the dehydration occurring upon binding is larger for H:R71A than for WT. This result suggests that the fluctuation of H:R71A in the free form is suppressed but the H:R71A undergoes structural rearrangement after binding by the induced-fit mechanism. The Δ*C*_*p*_ of L:R45A was similar to that of WT.

**Table 1:** Thermodynamic parameters of the interaction with antigen.

	T [K]	Δ*H* [kJ/mol]	Δ*C*_*p*_ [kJ/mol/K]
H:R45A	293	68.88 ± 0.21	−1.04 ± 0.03^*a*^
	298	−73.50 ± 0.15	
	303	−79.38 ± 0.23	
L:R71A	293	−99.50 ± 0.25	−2.85 ± 0.07^*a*^
	298	−113.40 ± 0.36	
	303	−130.29 ± 1.45	
WT	293		−1.4^*b*^

^*a*^Derived from *ΔH* values measured at 293, 298, and 303 K. ^*b*^Taken from ref. 14.

Next, we analyzed the thermal stability of H:R71A and L:R45A by differential scanning calorimetry (DSC) to gain insight into the mechanism by which the mutations increased the affinity. The Δ*H* value of the lysozyme-bound form of H:R71A and L:R45A was 302 and 313 kcal/mol, respectively (Supplementary Table 2, Supplementary Fig. 4). These values are larger than that of WT (247 kcal/mol), indicating that both mutants form more stable complexes as compared with WT. For H:R71A, the large change in Δ*C*_*p*_ and the large contribution of Δ*H* to binding suggest that formation of the stable complex is accomplished by tighter packing of the outer loop and CDR-H3, which are adjacent to H:Ala71. On the other hand, for L:R45A, where the mutated residue is involved in the V_L_ loop adjacent to the V_H_ domain (Supplementary Fig. 1), the small change in Δ*C*_*p*_ and the small contribution of Δ*S* shows that the structural rearrangement that occurs upon interaction with the antigen is decreased by the mutation. This is thought to be caused by a slight reorientation of the V_H_ and V_L_ domains of the free form to a more favorable binding orientation.

### Validation 1: Fluctuation of the affinity-increased mutants in the free form

**Figure 4:**
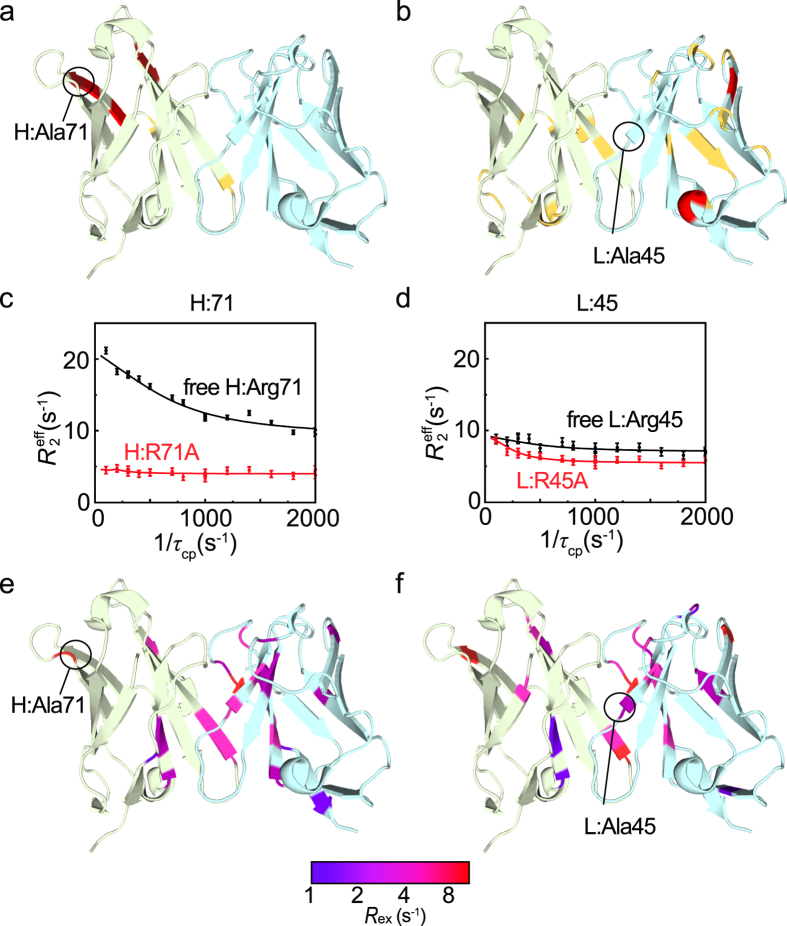
Mutants with increased affinity have fewer fluctuating residues. (**a**,**b**) Magnitude of the chemical shift changes caused by the H:R71A (**a**) and L:R45A (**b**) mutations shown in yellow for those greater than the average and in red for those greater than the average plus one standard deviation. (**c**) Relaxation dispersion profiles of H:R71 in free WT (black) and H:A71 in free H:R71A (red). (**d**) Relaxation dispersion profiles of L:R45 in free WT (black) and L:A45 in free L:R45A (red). (**e**,**f**) Amplitude of the *R*_ex_ rates for H:R71A (**e**) and L:R45A (**f**) mapped on the crystal structure of free HyHEL-10, as a continuous color scheme from purple to red. The V_H_ and V_L_ domains are shown in light green and light blue, respectively.

The HSQC spectra of H:R71A and L:R45A in their free form were similar to that of free WT, indicating that the overall structures of the mutants are similar to that of WT (Supplementary Fig. 3). For H:R71A, however, several residues adjacent to the mutated residue in the outer loop showed relatively large changes in chemical shift (Fig. 4a), suggesting that the conformational change induced by the Ala mutation is confined to the local area near the V_H_ domain. For L:R45A, chemical shift changes were observed in both the V_L_ and V_H_ domains (Fig. 4b), which supports the ITC data implying that the Ala mutation in L:R45A causes a reorientation of V_H_ and V_L_, leading to more favorable lysozyme binding.

To examine whether the mutations that we introduced altered the conformational fluctuations of HyHEL-10 as intended, we measured the *R*_2_ relaxation dispersions for each mutant (Fig. 4c,d). The exchange rate, *k*_ex_, for H:R71A and L:R45A was 753 ± 23 and 657 ± 15 s^−1^, respectively. These rates were slightly slower than that observed for the corresponding region in WT. For H:R71A, the relaxation dispersion profiles of several residues, including the mutated residue Ala71 and those in the outer loop and CDR-H3 (Gln3, Val24, Thr25, Ser28, Ser31, Trp36, Thr56, Ser68, and Asp72) became flat lines, meaning that no fluctuations of these residues were observed; however, relaxation dispersions in the V_L_ domain, which is distal to H:Ala71, were little influenced by the mutation (Fig. 4e). Together with the chemical shift changes observed for H:R71A, these results suggest that the H:R71A mutation changed the local conformation and fluctuations of the region adjacent to H:Ala71 in the V_H_ domain.

For L:R45A, no relaxation dispersions of residues at the interface of the V_L_ and V_H_ domains were observed (Fig. 4f), including His33, Gly84, Phe87, and Phe98 in the V_L_ domain, and Glu46 in the V_H_ domain. In addition, similar to H:R71A, relaxation dispersions of residues in the outer loop and CDR-H3 were not observed (Val24, Thr25, Ser28, Ser31, Trp36, and Asp72 in the V_H_ domain), suggesting that these residues are involved in the same fluctuation network that spreads over the both V_H_ and V_L_ domains. In particular, H:Glu46 is possibly an important residue for forming the fluctuation network. Indeed, the H:E46A mutant was not expressed in *Escherichia coli*, suggesting the importance of H:Glu46 in domain packing. The results indicate that suppressing fluctuations in the V_H_ domain is important for increasing the affinity of HyHEL-10.

### Validation 2: Comparison of fast timescale motion in the bound form

For bound WT HyHEL-10, relaxation dispersion provided little information about fluctuations on the millisecond timescale. Because fluctuations that are faster than the millisecond timescale cannot be detected by *R*_2_ relaxation dispersion, we analyzed and visualized fluctuations of the bound form on a faster timescale by using molecular dynamics (MD) simulation. We conducted a 130-ns MD simulation for the lysozyme-bound form of WT, H:R71A, and L:R45A. The overall structure did not change markedly during the simulation: the RMSD of the simulated structures from the average structure, which were aligned by Cα atoms, was less than 1.5, 1.2, and 1.4 Å for WT, H:R71A, and L:R45A, respectively. However, a local structural change was observed near the area of the mutated residue in both mutants.

**Figure 5:**
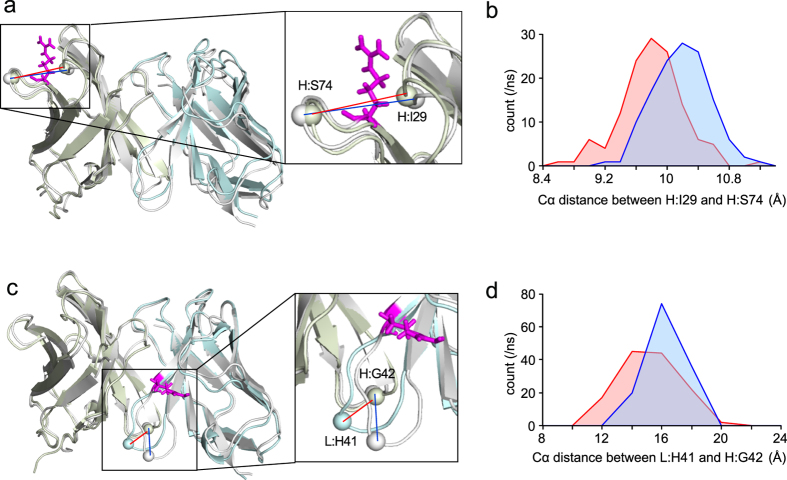
Mutation changes the distribution of intra-atomic distances in MD simulations. The V_H_ and V_L_ domain of the mutants are shown in light green and light blue, respectively. The structure of WT is shown in gray. (**a**) The distances between the Cα atoms of H:Ile29 and H:Ser74 in H:R71A and WT are shown as a red line and a blue line, respectively. The H:Arg71 residue is depicted as a magenta stick. (**b**) The distance distribution between the Cα atoms of H:Ile29 and H:Ser74 was compared between WT (blue) and H:R71A (red) to estimate the distance change in the CDR-H3 and outer loop. The *p* value was 4.4 × 10^−14^. (**c**) The distances between the Cα atoms of L:His41 and H:Gly42 in L:R45A and WT are shown as in (a). The L:Arg45 residue is depicted as a magenta stick. (**d**) The distance distribution between the Cα atoms of L:His41 and H:Gly42 was compared between WT (blue) and L:R45A (red) to estimate the distance change between the loops in the V_H_ and V_L_ domains. The *p* value was 2.4 × 10^−7^.

We measured the intra-atomic distances to examine the effect of the mutation. For H:R71A, the outer loop was a median value of 0.38 Å closer to CDR-H3 as compared with WT (Fig. 5a,b). For L:R45A, the adjacent V_H_ loop and V_L_ loop were a median value of 1.1 Å closer as compared with WT (Fig. 5c,d). These results indicate that the bound form of both mutants adopts a more packed conformation. The MD results suggest the possibility that the fluctuating H:Arg71 residue in WT disrupts the packing of the outer loop and CDR-H3 in the bound form (Fig. 5a) while the side chain of L:Arg45 flips between the V_H_ and V_L_ domains to disrupt their packing. For L:R45A, the V_H_ and V_L_ domains are more packed in the bound form (Fig. 5c,d). These data support the results of the ITC and DSC experiments showing an increase in the stability of the antigen-bound form of HyHEL-10.

## Discussion

We have described a method to increase the affinity of an antibody by regulating or “editing” its conformational fluctuation. We have set criteria to select specific residues in non-CDR regions that are distant from the antigen–antibody interface as candidates for mutation among the fluctuating residues detected by relaxation dispersion. Via this approach, we have succeeded in increasing the affinity of HyHEL-10 for lysozyme by more than 10-fold through a single Ala point mutation of a fluctuating residue. This increase in affinity is comparable to that achieved by molecular evolution methods targeting CDR residues, where the affinity may be increased by 10–20-fold in a single cycle^8^. To our best knowledge, this is the first study to show that the affinity of an antibody can be increased by rationally targeting the fluctuations of non-CDR regions.

NMR is one of the most powerful tools to analyze conformational fluctuations quantitatively, especially because it provides site-specific information. Although several prominent studies of antibodies using NMR, including those of conformational dynamics, have been reported^17–21^, studies on microsecond to millisecond fluctuations of the peptide backbone of antibodies have been scarce. Our study has shown that relaxation dispersion is suitable for detecting and quantifying fluctuations in free HyHEL-10 on the millisecond timescale. Although fluctuations of lysozyme-bound HyHEL-10 are barely observed by relaxation dispersion, MD simulation can be utilized for analyzing the motions of the bound form on the nanosecond timescale.

How do mutations of residues fluctuating far from the antibody–antigen interface increase the affinity? The relaxation dispersion experiments showed that fluctuations in the V_H_ domain are suppressed in the mutants; such conformational suppression will be important for packing of the antibody upon antibody–antigen interaction, resulting in an increase in affinity. A previous crystallographic study suggested that the V_H_ domain in WT is flexible, enabling structural adjustment upon binding^3^. Our mutation studies suggest that this flexibility in the outer loop may be an unfavorable factor in antigen binding. Together with the MD data, we show that motions unfavorable for binding occur in the V_H_ domain of WT. Removing the unfavorable fluctuations by a point Ala mutation leads to a rearrangement of intra-molecular interactions. The relaxation dispersion data also suggest that the fluctuation suppressed at the mutated residue is transmitted to distal regions, including the antibody–antigen interface^22^.

In summary, we have described a method of “fluctuation editing” to specifically detect and engineer intramolecular fluctuations. Although previous studies have reported the functional modification of proteins through changes in fluctuation^23–25^, our study is the first to propose a systematic experimental method to detect and engineer conformational fluctuations that are important for distal regulation. Via our method, we showed that mutation of a large residue to Ala decreases the number of the fluctuating residues in the antibody and that Ala mutation of large fluctuating residues is effective for increasing antibody–antigen affinity. Mutations of other residues selected on the basis of alternative criteria might further increase antigen affinity by a different mechanism. Furthermore, it would be worth combining our method with other protein-engineering methods, such as phage display or computer-assisted rational design^26^. Typical studies using phage display have focused on residues at the antibody–antigen interface^2–6, 10, 13, 14^. To apply phage display to non-CDR regions, however, it is necessary to set a criterion to specify a target area for molecular evolution, because of the high number of candidate residues in non-CDR regions. In this regard, an advantage of our fluctuation editing method is that it can narrow down the candidate residues in non-CDR regions that are potentially important for antigen binding.

Recent developments in NMR and isotope-labeling methods have enabled us to study the structure and dynamics of very large proteins with masses as large as 1 MDa^27^. For IgG, Arbogast *et al*. recently reported that Fc and Fab fragments are amenable to NMR measurements, at least for IgG1κ. Therefore, it is highly likely that our method can be applied directly to Fc and Fab^19^.

## Methods

### Protein expression and purification

The cDNA of HyHEL-10^14^ was cloned into a pET22a vector. [^15^N]- and [^13^C,^15^N]-HyHEL-10 proteins were expressed as inclusion bodies in BL21 (DE3) cells grown in M9 minimal medium. When the OD_600_ of the cell culture reached 1.2–1.5 at 37 °C, isopropylthio-b-D-galactoside (IPTG) was added to the medium at a concentration of 1 mM to induce protein expression. The protein-containing cells were lysed and centrifuged. The pellet was washed three times in 50 mM Tris-HCl buffer with sonication. The HyHEL-10 Fv fragment was dissolved in 6 M guanidine hydrochloride (Gdn-HCl) buffer and prepared at a concentration of 5–6 mg/ml and dialyzed against refolding buffer (100 mM Tris-HCl, 0.5 mM GSSG, and 5 mM GSH)^14^ over night. The dissolved protein was purified using lysozyme-immobilized affinity column as described previously^13^. After affinity chromatography, the obtained HyHEL-10 was further purified with size exclusion chromatography under the condition of 150 mM phosphate buffer with 300 mM NaCl. The final yield of the protein was 10 mg/l of *Escherichia coli* culture.

### NMR methods

Three-dimensional spectra of HNCO, HN(CA)CO, HNCA, HN(CO)CA, HNCACB, HN(CO)CACB, and^15^N NOESY-HSQC were measured on an AVANCE DRX600 spectrometer (Bruker BioSpin) for sequential assignments of the backbone^1^H,^13^C, and^15^N chemical shifts^28^ of free and bound HyHEL-10 using protein dissolved at 0.8–0.9 mM in NMR buffer (95% H_2_O/5% D_2_O, 20 mM PBS [pH 7.4], 143 mM NaCl). NMR data were processed and analyzed as previously described^29^.

^15^N effective *R*_2_ relaxation rates were measured at 37 °C on AVANCE DRX600 and AVANCE DMX750 spectrometers (Bruker BioSpin) using the^1^H continuous-wave decoupled Carr-Purcell-Meiboom-Gill (CPMG) pulse sequence^30^. Effective *R*_2_ rates were calculated as described previously^11^. For L:R45A, non-uniform sampling and the SIFT method^31^, were used to shorten the experimental time because the free form of this mutant was not stable enough to measure a full set of *R*_2_ relaxation spectra in the standard way. Relaxation dispersion data whose *R*_2_ values changed by < 1 s^−1^ over the entire range of τ_cp_ were excluded.

The relaxation dispersion curves were fitted globally by using the program GLOVE^12^ with a certain cluster in which neighboring residues were assumed to fluctuate at the same exchange rate. The Carver and Richards equation and the Luz and Meiboom equation were used to fit the relaxation dispersions. The Carver and Richards equation is appropriate for a two-state exchange model (major ↔ minor) in the intermediate or slow exchange regime, and a curve fit to this equation yields the population-average intrinsic transverse relaxation rate (*R*_2_^0^), the exchange rate *k*_ex_, the chemical shift difference between states (Δ*ω*), and the populations of the major and minor states (*p*_major_, *p*_minor_). The Luz and Meiboom equation is valid only for the fast exchange regime, and a curve fit to this equation yields *R*_2_^0^, *k*_ex_, and *p*_major_ *p*_minor_Δω^2^. Using these two equations, we tested several global fits for various clusters involving different groups of residues. The fitting quality was compared among different fits by the reduced χ^2^ value (χ^2^ divided by the degree of freedom) and *F* test to determine the appropriate exchange regime and clusters to describe the fluctuations observed by relaxation dispersion. The Luz and Meiboom equation fitted better for H:R71A and L:R45A, whereas the Carver and Richards equation was suitable for fitting free WT data.

### Isothermal titration calorimetry measurements

Protein samples were dialyzed against PBS (20 mM PBS [pH 7.4], 143 mM NaCl), and the concentrations of the antibody and lysozyme were adjusted to approximately 30 μM and 2 μM, respectively. Thermodynamic analysis was performed to investigate the interaction between lysozyme and antibody using an VP-ITC isothermal titration calorimeter (MicroCal). The antibody was titrated against lysozyme 25 times with the amount of 10 μl and the duration of 20 seconds for each titration. ITC experiments were carried out at 20, 25, and 30 °C under otherwise identical conditions. The experimental data were baseline-corrected and subjected to *K*_A_ calculation by using the software package ORIGIN for ITC(MicroCal) as described previously^14^.

### Differential scanning calorimetry measurements

Protein samples were dialyzed against PBS (20 mM PBS [pH 7.4], 143 mM NaCl), and the concentrations were adjusted to approximately 2 mg/ml. To examine the heat stability of the proteins, heat capacity curves were obtained by an ultrasensitive VP-DSC scanning microcalorimeter (MicroCal) at a heating rate of 1 K/min with a sample cell volume of 0.5 ml. The obtained data were baseline-corrected and subjected to deconvolution by using the software package ORIGIN for DSC (MicroCal) as described previously^32^.

### Molecular dynamics simulation

Three-dimensional coordinates of WT HyHEL-10 were obtained from the PDB database (PDB ID: 1C08)^3^. Models of the H:R71A and L:R45A mutants were constructed using the “mutagenesis” function implemented in PyMOL^33^. Water molecules that were resolved in the crystal structure were included in the simulations. Preparation of initial structures for the MD simulation, energy minimization, and heating was performed as described previously^34^. The minimization and equilibration runs were conducted by using GROMACS version 5.0.4^35^. Each production run was carried out for 130 ns, maintaining the temperature at 300 K and the pressure at 1.0 × 10^5^ Pa. Distances between the two atoms were measured for the structures extracted every nanosecond from the 130-ns simulation trajectory using the program CPPTRAJ^36^. The ASA of the V_H_ and V_L_ domains was calculated by using PyMOL^33^.

## Electronic supplementary material

Supplementary figures 1-4, Supplementary table 1-2
