# Supplementary material for: Elucidation of potential sites for antibody engineering by fluctuation editing
Source: Sci Rep. 2017 Aug 30;7:9597. doi: 10.1038/s41598-017-10246-9 (PMC5577056; doi:10.1038/s41598-017-10246-9)
Supplement: Supplementary file 1 — Supplementary figures 1-4, Supplementary table 1-2 [file 41598_2017_10246_MOESM1_ESM.pdf]

Elucidation of potential sites for antibody engineering by fluctuation editing

Saeko Yanaka[1,†], Yoshitaka Moriwaki[2], Kouhei Tsumoto[3,4], Kenji Sugase[1,¶]

[1]Bioorganic Research Institute, Suntory Foundation for Life Sciences. [2]Department of Biotechnology and Agricultural Bioinformatics Research Unit, Graduate School of Agricultural and Life Sciences, The University of Tokyo. [3]Department of Bioengineering, Graduate School of Engineering, The University of Tokyo. [4]Laboratory of Medical Proteomics, Institute of Medical Science, The University of Tokyo. Correspondence should be addressed to K.S. (sugase@moleng.kyoto-u.ac.jp)

[†]Currently Department of Life and Coordination-Complex Molecular Science, Biomolecular Functions, Institute of Molecular Science, National Institute of Natural Sciences.
[¶]Currently Department of Molecular Engineering, Graduate School of Engineering, Kyoto University.

Supplementary Table 1 Fluctuaing residues categorized by our criteria

Small residues	L:S7(72) [a], L:S54(100), L:G66(73), L:S74(46), L:G84(35), L:S91(33), L:T97(25),L:G99(7), L:G100(100), L:G101(13), H:V24(10), H:T25(85), H:S28(71), H:S31(91), H:T57(48), H:A91(0)
CDR	L:Y96(20), H:Y58(59)
Large BSA	L:F98(0), H:C22(0), H:W36(0), H:L45(11), H:Y94(2), L:Y86(0), L:H34(4), L:L47(0.0)
Selected residues	L:R45(69), H:Q3(100), H:N76(29), H:D27(69), H:R44(97), H:E46(22), H:R71(54), H:D72(46)

[a] The percentage of accessible surface area of residues are shown in parentheses.

Supplementary Table 2 DSC parameters

	DSC ΔH [kcal/mol]	
	free form	complex
L:R45A	120 ± 1	302 ± 2
H:R71A	199 ± 1	313 ± 2
WT	194 ± 1	247 ± 3

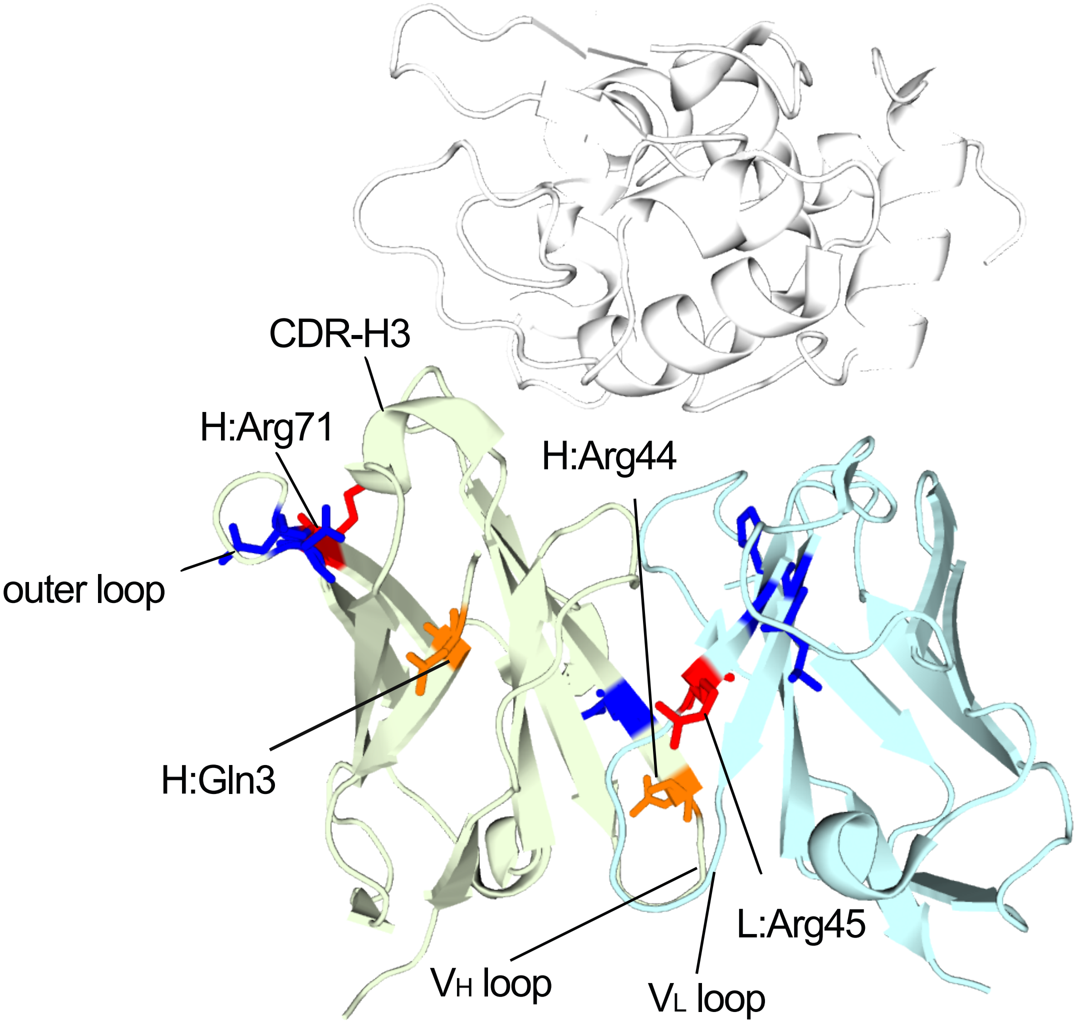

Supplementary Fig. 1 The structure of HyHEL-10.

The residues in which a single alanine-mutation was introduced in this study are colored as the following. Red: the mutants increased the affinity. Orange: the mutants were successfully refolded but the affinity increase could not be observed. Blue: the mutants could not be obtained. The V_H and V_L domainsare shown in light green and light blue, respectively. Lysozyme is colored as gray.

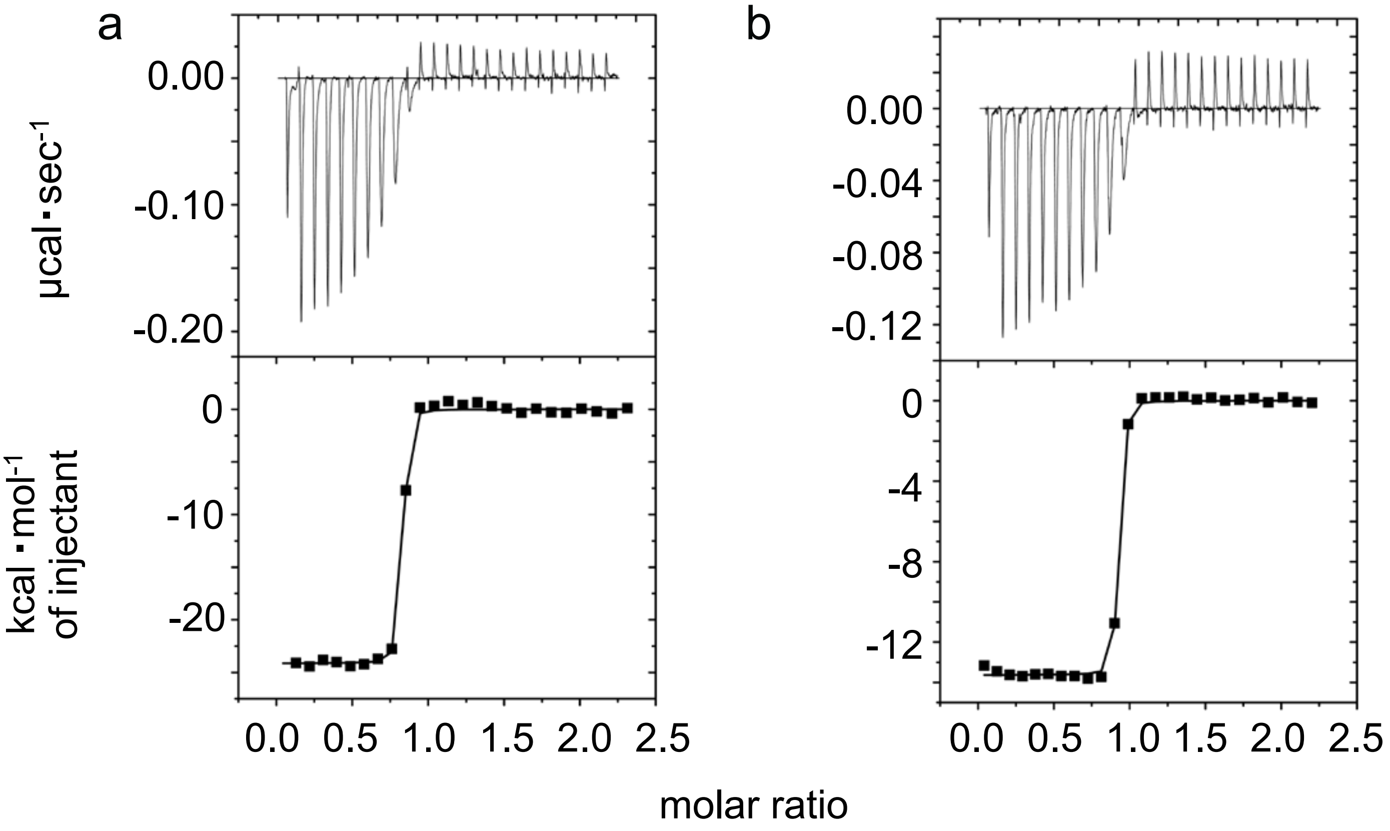

Supplementary Fig. 2 The ITC profiles of H:R71A and L:R45A titrated against lysozyme.

The measurements were conducted at 20°C for H:R71A (a), and L:R45A (b).

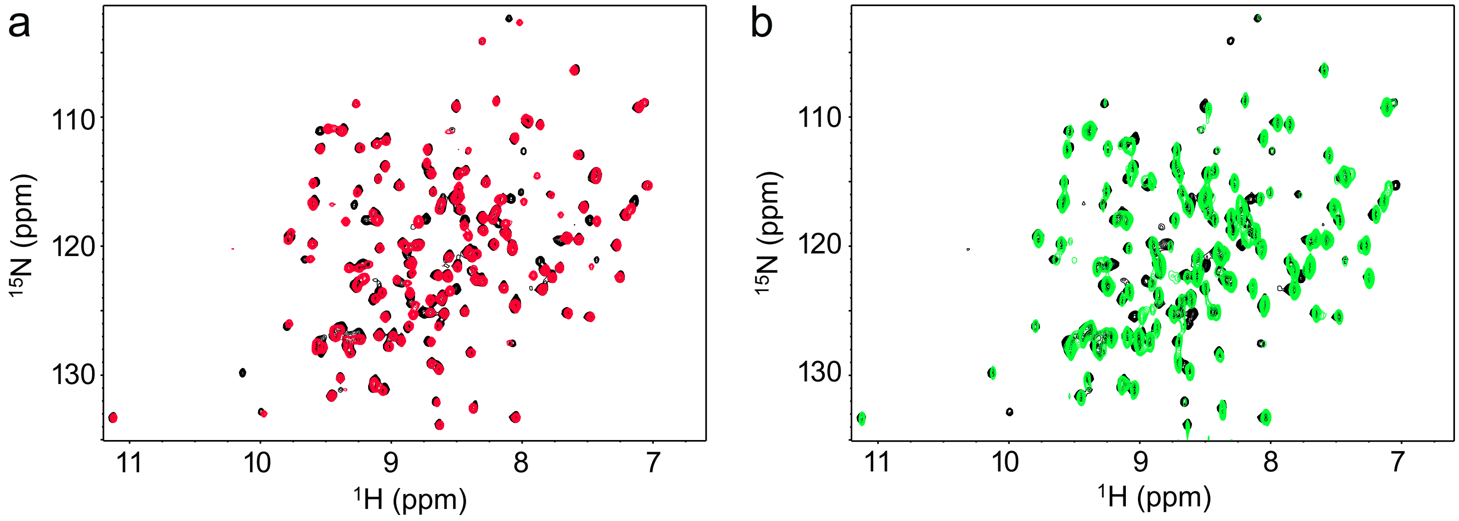

Supplementary Fig. 3 Comparison of the HSQC spectra between WT and mutants.

(a) HSQC spectra of WT and H:R71A in the free form are shown in black and red, respectively. (b) HSQC spectra of WT and L:R45A in the free form are shown in black and green, respectively.

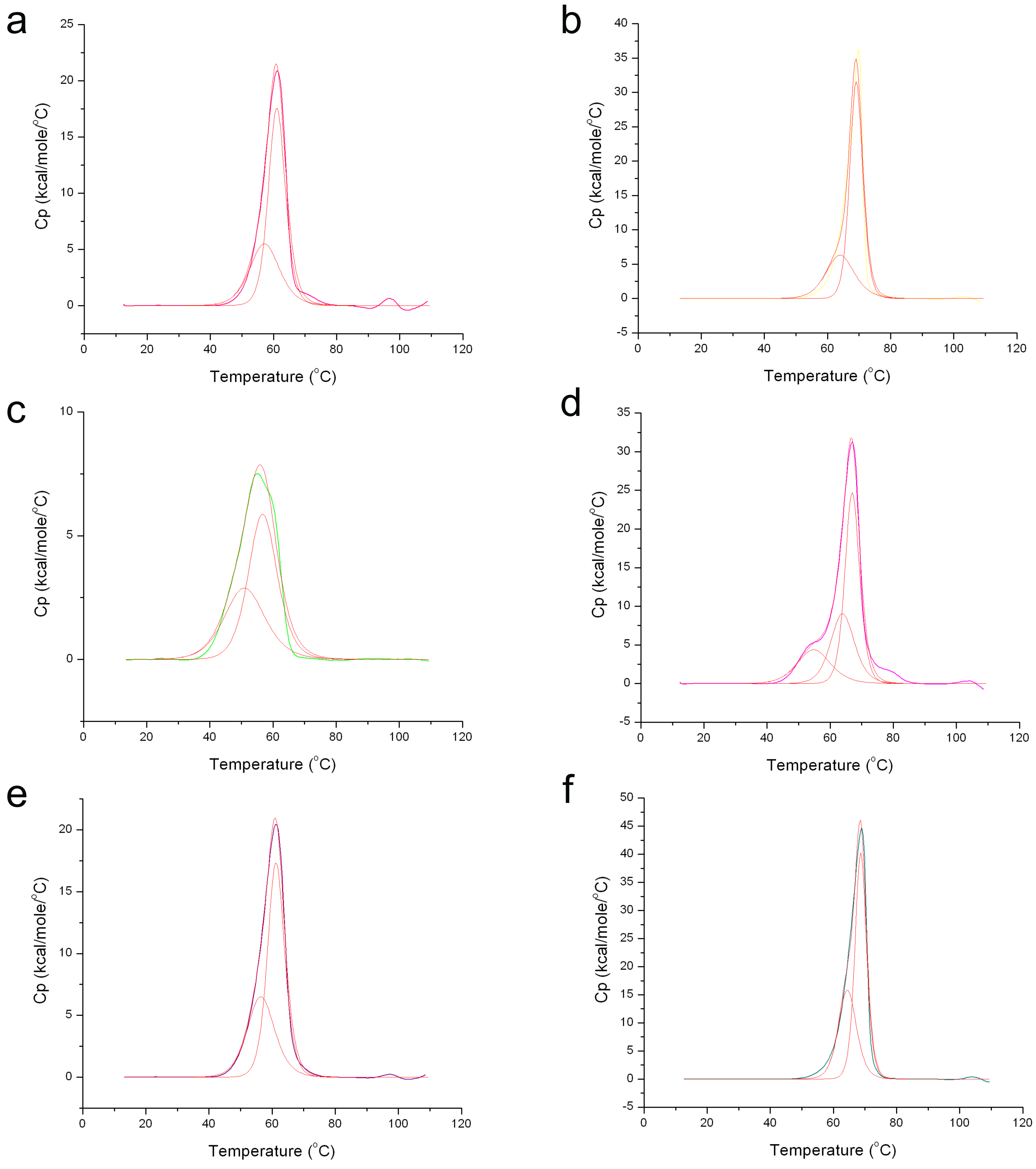

Supplementary Fig. 4 DSC profiles of HyHEL-10 and its mutants.

The DSC curves are shown for (a) WT HyHEL-10, (b) WT HyHEL-10 in complex with lysozyme, (c) L:R45A, (d) L:R45A in complex with lysozyme, (e) H:R71A, (f) H:R71A in complex with lysozyme. The red curves show the deconvolution of the expetimental curves.
